# Epidemiology of respiratory syncytial virus in a German hospital before, during and after the COVID-19 pandemic – a real world monocentric analysis over ten years

**DOI:** 10.3389/fped.2026.1771759

**Published:** 2026-05-04

**Authors:** Svenja Loerch, Frank Erdnüß, Beate Frerich, Thorsten Reineke, Wolfgang Kamin

**Affiliations:** 1 Pediatrics, Johanniter Medical Center Hamm, Hamm, Germany; 2 Pharmacy, University Medical Center, Johannes Gutenberg-University Mainz, Mainz, Germany; 3 Department of Economics, Baden-Wuerttemberg Cooperative State University, Karlsruhe, Germany

**Keywords:** COVID-19, epidemiology, respiratory tract infection, RSV, vaccination, single center study

## Abstract

**Background:**

RSV infection affects mostly infants and induces a substantial economic and medical burden for our health care systems. To implement effective preventive strategies against RSV the knowledge about its distinct seasonality is crucial.

**Methods:**

We retrospectively studied the epidemiology of RSV infection in pediatric patients admitted to a German hospital over a period of ten years by comparing their clinical data. Inclusion criterion was a laboratory confirmed RSV infection with diagnosis of bronchitis, bronchiolitis, or pneumonia. For analyses six pre-COVID years were cumulated and compared to the pandemic and post-pandemic seasons. Statistical methods included descriptive measures and exploratory tests for comparing seasons (i.e., Fisher's exact test).

**Results:**

Overall, data of 1,220 children below 12 years of age (including 1,040 below 2 years) could be analyzed. The pre-COVID RSV seasons started in December and peaked in January/February. During the pandemic we detected no case in 2020/21. Season 2021/22 started in August and peaked in October, whereas the first post-pandemic season 2022/23 started in October and peaked in November. The need for intensive care and oxygen supply was significantly increased in 2022/23 compared to 2021/22. Moreover, bacterial superinfection and antibiotic treatment appeared to be significantly more frequent, also in comparison to the pre-COVID seasons.

**Conclusion:**

Our study shows that the usual RSV epidemiology abruptly changed with the start of the COVID-19 epidemic. After a loss of severe RSV associated disease in 2020/21 a catch up effect could be notified in hospitalized patients in the following seasons, ending up in an almost normal season 2024/25. Future RSV monitoring is strongly recommended to calculate the hospital necessary resources, since vaccination programs, i.e., nirsevimab for infants and maternal vaccination during pregnancy, have already been started in Germany.

## Introduction

Affecting annually around three million children worldwide, respiratory syncytial virus (RSV) entails a substantial medical and economic burden on health care facilities and thereby showing a distinct epidemic seasonal pattern (1, 2). Infants are the mainly affected age group with a hospitalization rate of about 2% in healthy term-born infants (3). In Germany, RSV results in high numbers (around 20,000 each year) of hospitalizations in infants with severe acute respiratory infection (i.e., bronchitis, bronchiolitis, pneumonia) (4) and also affects older children and the elderly (5). The knowledge about the seasonality of RSV infections and the disease severity is a prerequisite for preventive strategies and new treatment options against RSV infection (6). Recently, various studies have shown changing patterns of RSV infection in the last few years. Mainly, a shift of the annual onset of RSV infection and different disease severity were found (7–12). Both findings seem to be interrelated to the COVID-19 pandemic and its non-pharmaceutical interventions, i.e., mandatory public health measures as the wearing of face-masks and increased hygienic behavior (13, 14). As expected, patterns vary between countries around the world dependent on climatic factors and strictness of infection preventions (15). While a shift of the onset and the mainly affected age groups, i.e., hospitalized children during the pandemic were older, are observed in most studies, the data on disease severity is inconsistent (16, 17).

Since further pandemics are likely in the future, we should be prepared to protect our vulnerable cohorts at the best. Prophylactic interventions against RSV infection include new vaccines as well as monoclonal antibodies (18–22). Currently, passive immunization with Palivizumab is rare in Germany, since it is only approved for risk groups and pre-term infants. Wick et al. (23) report for the birth cohorts 2015–2019 that less than 2% of infants received at least one dose of palivizumab. With the new monoclonal antibody nirsevimab, which was approved by the European Medicines Agency (EMA) in October 2022 and by the US Food and Drug Administration (FDA) in July 2023 for all infants and young children (24), numbers of passive immunization will probably increase. Recent studies found an effectiveness of nirsevimab of 93% against RSV-associated hospitalization (25, 26), but the US study reports immunization rates in infants of only 15%; this is estimated to low for a substantial reduction of hospitalization rates (26). Another new product for prevention is the RSV prefusion F vaccine (RSVpreF), which can be administered to the elderly and also to pregnant women. By transplacental antibody transfer it is able to protect infants in their first RSV season. A systematic review reports promising results for this strategy, although some concerns about safety outcomes still remain (27). These medicines have yet been licensed only in developed countries, but since the vast majority of worldwide RSV infections occur in low-income countries, access to these vaccines should be extended (28).

All preventive measures should be target-oriented, i.e., pregnant women (22), infants, and infants with certain co-morbidities (29). Moreover, the right timing of any intervention is most important (29), leading back to the current challenge: We need to analyze all available data on RSV patterns connected with the COVID-19 pandemic very thoroughly.

In Germany the first SARS-CoV-2 infection was registered on January 27th 2020. On March 22nd the government imposed the first lockdown in Germany, including all the particular interventions well enough known. This policy certainly influenced other infectious diseases, mirrored amongst others in the study results cited above (30). Our analyses aim to contribute to the already published data by providing clinical and epidemiological information on RSV infections from a children's hospital before, during and after the COVID-19 pandemic. We retrospectively compared clinical data of 1,220 children hospitalized with RSV during the years 2015 to 2025 and thereby gained insight into the specific infection dynamics. The results could help to identify target populations and the right time for prophylaxis in the future.

## Materials and methods

### Study design and population

We retrospectively evaluated clinical data of all pediatric patients with laboratory confirmed RSV infection hospitalized in the children's hospital of the Lutheran Hospital in Hamm, Germany, in the years 2015 to 2025. Inclusion criteria were laboratory confirmed RSV infection with diagnosis of bronchitis, bronchiolitis, or pneumonia.

Under the general assumption that severe RSV illness only occurs in patients under 2 years of age, only those children with appropriate symptoms were tested until 2020. Afterwards we extended testing to all our age groups. For a consistent analysis we excluded children of two years and older.

### Test material

To detect RSV infection we performed two different antigen tests from nasal/pharyngeal swab: until March 31st 2018 the BinaxNOW™ RSV test (Abbott GmbH, Wiesbaden, Germany) and afterwards the ID NOW™ RSV PCR test (Abbott GmbH, Wiesbaden, Germany) until 31.12.2024. Since January 2025 we now use the real time PCR test Savanna (QuidelOrtho Corporation, San Diego, USA - Panel RSV, Influenza A/B SarsCov2).

### Data collection and treatment

In addition to the test results we gathered all data from our hospital charts and the discharge letters, which we transferred to our standardized documentation form for further statistical analyses. Evaluated clinical data included the following parameters: Prematurity, birth weight, Co-morbidities, Prophylaxis (Palivizumab or Nirsevimab), duration of hospital stay, diagnosis (bronchiolitis, bronchitis, and pneumonia), x-ray, need of intensive care, oxygen needed (The general cut off for oxygen supplementation was below 90%), ventilation needed, fever, and medication.

We used internal standard operation procedures (SOP) for treatment of RSV (monitoring, need of oxygen supplementation, inhalation strategies, i.v. hydration/nasal tube feeding), which was changed over the years due to new guidelines. The changes especially comprised the inhalation strategies, i.e., changes in using Salbutamol, Adrenalin or NaCl 3% as preferred medium.

### Statistical analysis

The six pre-COVID years (2015–2020) were cumulated in all analyses and compared to the pandemic RSV seasons 2020/21 and 2021/22 and the post-COVID seasons 2022/23, 2023/24, and 2024/25. Descriptive measures were used to describe all observed variables – counts and ratios for qualitative variables as well as mean, standard deviation, minimum, median and maximum for quantitative measures. In order to compare different seasons Fisher's exact test as well as Wilcoxon's Rank-Sum-test was used. Resulting *p*-values are interpreted in an exploratory manner assuming a level of significance of alpha=0.05. Due to the exploratory nature of the tests an adjustment of *p*-values is not necessary. Graphical visualizations of the numerical results are presented additionally. All analyses and charts were produced using R (version 4.2.3) with RStudio (version 2023.03.0).

## Results

### Study population

Our retrospective study of RSV epidemiology included data of 1,220 patients, including 1,040 children below 2 years, admitted over 10 years to one hospital in Germany. Demographics and important clinical parameters are given in Table 1. The check for comorbidities, including prematurity, in the patient population revealed, that they were rather rare (below 5%) and randomly distributed. There was no hint for any cumulation.

**Table 1:** Demographics and clinical data of the study population.

SEASON	2015–20	2021/22	2022/23	2023/24	2024/25
	(*N* = 398)	(*N* = 263)	(*N* = 229)	(*N* = 205)	(*N* = 125)
AGE (days)					
Mean (sd)	249.54 (389.08)	416.23 (455.94)	347.03 (433.14)	371.31 (507.69)	774.09 (1,258.49)
Median	142.00	227.00	155.00	178.00	374.00
AGE-GROUP					
a) < 1 yr	83.42% (*n* = 332)	61.22% (*n* = 161)	72.49% (*n* = 166)	69.76% (*n* = 143)	49.60% (*n* = 62)
b) 1 to below 2 yrs	10.05% (*n* = 40)	19.39% (*n* = 51)	10.04% (*n* = 23)	15.61% (*n* = 32)	24.00% (*n* = 30)
c) 2 yrs and older	6.53% (*n* = 26)	19.39% (*n* = 51)	17.47% (*n* = 40)	14.63% (*n* = 30)	26.40% (*n* = 33)
GENDER					
Female	39.70% (*n* = 158)	45.63% (*n* = 120)	47.60% (*n* = 109)	44.39% (*n* = 91)	41.60% (*n* = 52)
DURATION (days)					
N	398	263	227	205	125
Mean (sd)	6.51 (12.77)	3.83 (2.86)	4.32 (4.12)	4.16 (3.03)	3.99 (2.39)
Median	4.00	3.00	4.00	3.00	3.00
DIAGNOSIS					
Bronchiolitis	61.31% (*n* = 244)	67.68% (*n* = 178)	70.74% (*n* = 162)	63.41% (*n* = 130)	34.40% (*n* = 43)
Bronchitis	19.60% (*n* = 78)	20.91% (*n* = 55)	16.16% (*n* = 37)	20.49% (*n* = 42)	48.00% (*n* = 60)
Pneumonia	18.59% (*n* = 74)	11.03% (*n* = 29)	13.10% (*n* = 30)	16.10% (*n* = 33)	17.60% (*n* = 22)
RSV-INFECTION					
Nosocomial	3.77% (*n* = 15)	0.76% (*n* = 2)	0.44% (*n* = 1)	0.49% (*n* = 1)	0.00% (*n* = 0)
x-RAY					
Yes	33.42% (*n* = 133)	14.45% (*n* = 38)	23.14% (*n* = 53)	19.51% (*n* = 40)	23.20% (*n* = 29)
FEVER					
Yes	56.28% (*n* = 224)	54.37% (*n* = 143)	58.95% (*n* = 135)	69.76% (*n* = 143)	65.60% (*n* = 82)
BACTERIAL SUPERINFECTION					
Yes	13.07% (*n* = 52)	10.65% (*n* = 28)	22.27% (*n* = 51)	24.39% (*n* = 50)	27.20% (*n* = 34)
MEDICATION					
Antibiotics	13.07% (*n* = 52)	11.03% (*n* = 29)	21.83% (*n* = 50)	24.39% (*n* = 50)	26.40% (*n* = 33)

Since inhalation strategies for treatment of RSV varied during the study period, i.e., patients used Salbutamol, Adrenalin or NaCl 3% as preferred medium, it is important to state that we didn't notice significant changes in the outcome.

### Epidemiology of RSV

**Figure 1:**
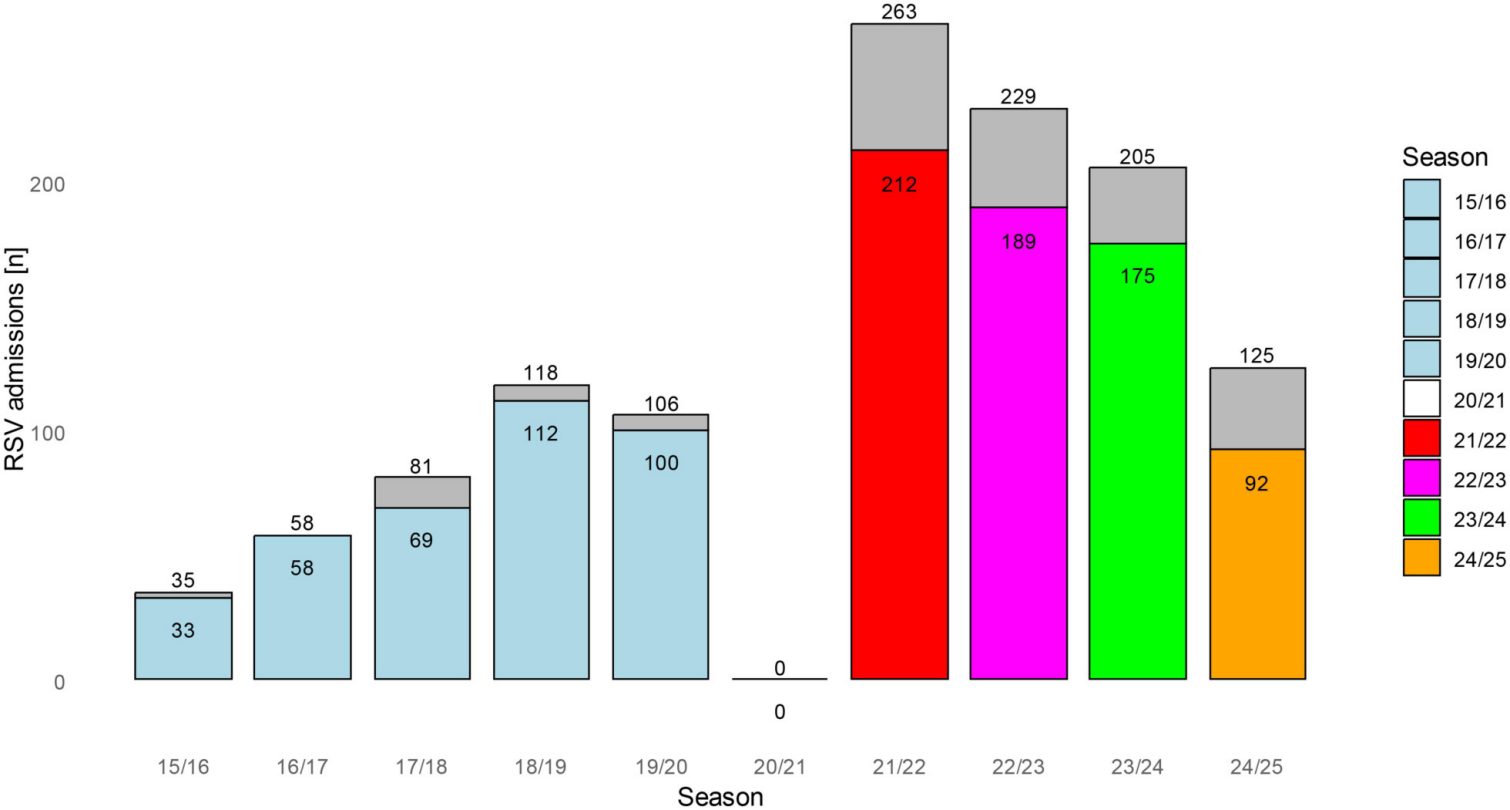
Number of patients with confirmed RSV infection admitted to our clinic over 10 years. No case was detected during the pandemic in season 2020/21 between April 9th 2020 and July 23rd 2021. The grey parts of the columns indicate the proportion of children 2 years and older.

In the pre-COVID years 2015–2020 RSV cases in our hospital ranged between 35 and 118 cases per season (Figure 1). After the start of the COVID-19 pandemic (first case in Germany was reported on January 27th 2020) RSV admissions ceased completely for more than a year, directly after the first lock-down on March 22nd. The last patient was admitted on April 9th 2020. Subsequently, we had no patients with confirmed RSV infection admitted to our clinic until July 23rd 2021. Adjacent numbers of RSV positive patients increased dramatically until December 2021. In the following seasons patient numbers returned stepwise to almost the same level as in the pre-COVID years (Figure 1).

**Figure 2:**
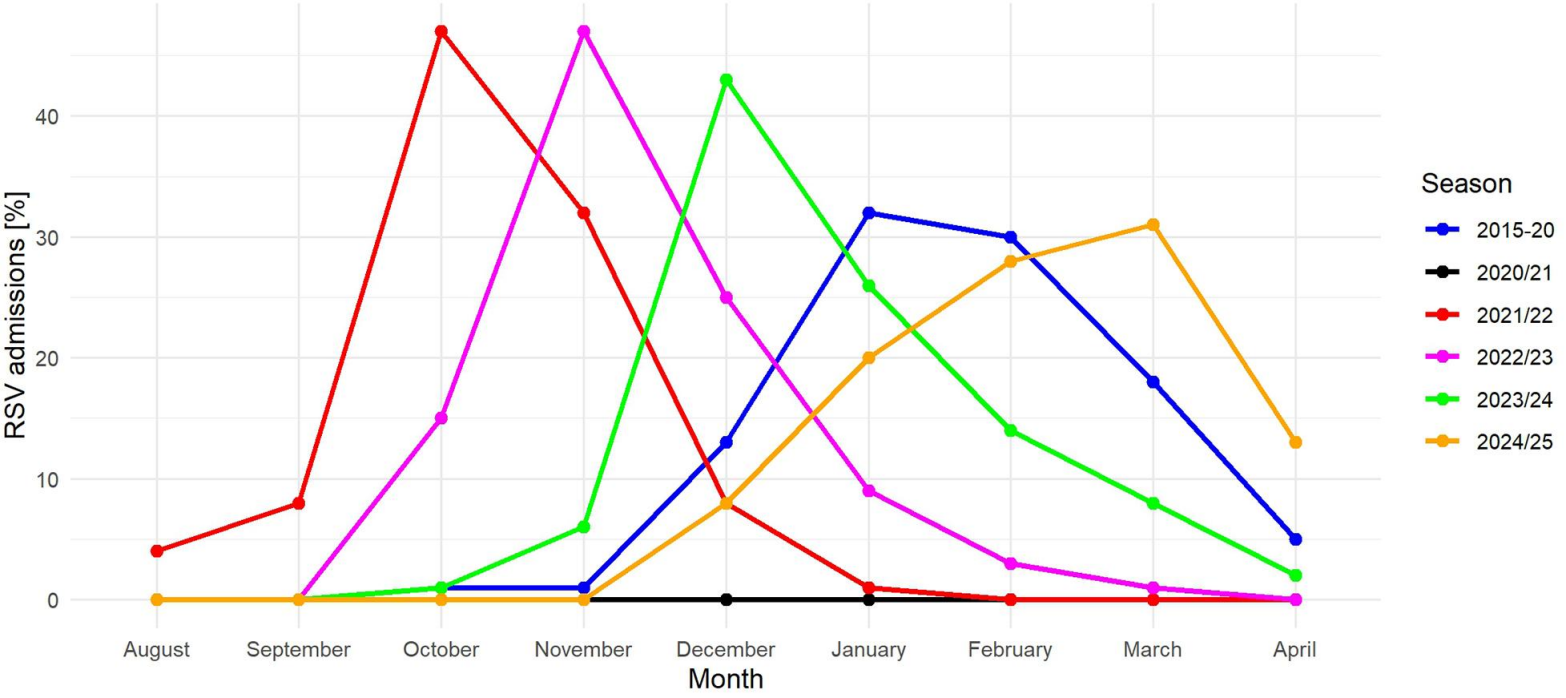
Seasonality of RSV in children below 2 years of age before, during and after the COVID-19 pandemic. Pre-COVID seasons (2015-20) are cumulated. No cases were detected in season 2020/21.

Concerning the start of RSV seasons, we detected a shift: In the pre-COVID years seasons started in October (December), reached their peak in January/February and ceased in May. In contrast, during the pandemic we detected the first RSV case in July 2021, the most admissions in October and the last patient already in December 2021. Afterwards, seasonality returned to normal with a start in October/November and an end in March/April. However, peaks of the post-COVID seasons 2022/23 and 2023/24 were still earlier than in pre-COVID years. In contrast, the most recent RSV season peaked in March 2025 (Figure 2).

### Severity of RSV infection

When focusing on the most affected age group below 2 years, we found significantly higher incidences in 2021/22 and less need for intensive care compared to pre-COVID seasons. The cumulatively analyzed pre-COVID cohort, consisting of 398 cases appearing from 2015 to 2019, shows a significantly longer duration of hospital stay and x-rays were performed more frequently compared to the COVID-cohort from 2021/22, consisting of 263 cases.

**Figure 3:**
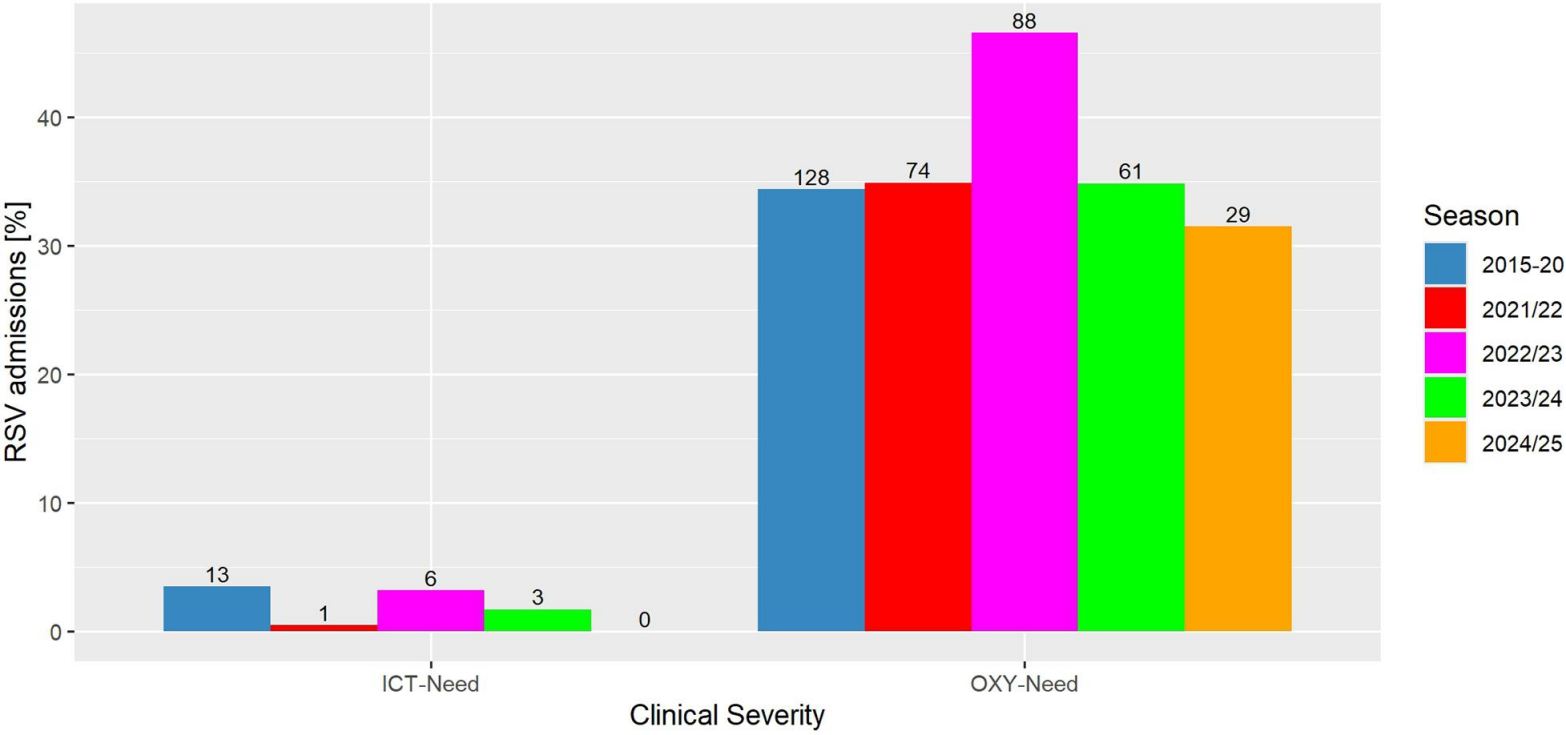
Severity of RSV infections before, during and after the COVID-19 pandemic. Absolute numbers of patients are given on each column. ICT, Intensive care treatment; OXY, Oxygen. Pre-COVID seasons (2015-20) are cumulated. No cases were detected in season 2020/21.

Looking at further severity parameters, we detected more cases of pneumonia and a significantly increasing need of intensive care in the first post-pandemic season (2022/23) compared to 2021/22 (*p* = 0.05) (Figure 3). The need of oxygen significantly increased compared to 2021/22 (*p* = 0.05) and also in comparison to the pre-COVID years (*p* = 0.03) (Figure 3). Also, the number of bacterial superinfections, laboratory indicated by CRP and leucocytes, increased significantly compared to the pandemic season (*p* = 0.0001) and also in comparison to the pre-COVID years (*p* = 0.003). Accordingly, administration of antibiotics was significantly increased in this first post-COVID season.

## Discussion

This real-world analysis in our hospital (monocentric database) confirms the well-known RSV epidemiology during the pre-COVID years 2015–2020, i.e., RSV seasons start in November, peak in January/February and cease in April (5). Concerning RSV incidence we observed an increasing trend in pre-COVID years, in line with the incidences reported by Schönfeld et al. (31) for Saxony (Germany). With the beginning of the first lockdown because of the COVID-19 pandemic on March 22nd 2020, the RSV wave abruptly ended. The last patient was admitted to our hospital on April 9th 2020, followed by a complete shortfall of critical ill RSV patients until Juli 23rd 2021. During the 2020/21 season, we were unable to detect any RSV cases among the children admitted as inpatients. This is consistent with the published literature (32–38) and can be attributed to the generally applicable hygiene measures that were in place during the time of the pandemic (13, 14, 39, 40). It shows that wearing masks, good hand hygiene and social distancing are effective measures against the spread of contagious diseases, also to protect our health care personnel. In contrast, Schönfeld et al. (31) report more than 4,500 laboratory confirmed RSV-cases for Saxony (Germany) in 2020/21 in all age groups and Manno et al. (41) also found RS-virus still present in the community in 2020/21, when analyzing viral respiratory infections in older age groups in Southern Italy. Li et al. (42) found that RSV pattern in Sweden was influenced by influenza virus, indicating an interplay between different respiratory viruses and Zhao et al. (43) report viral shifts among respiratory pathogens in China during the COVID-19 epidemic. An interesting aspect is to observe whether we will see such a viral shift also in the future, i.e., different respiratory viruses may fill the gap of RSV after vaccination takes place.

The very unusually early start of the RSV season in 2021/22 after the relaxation of hygiene measures suggests that the lack of relative immunity after the missing season led to the virus encountering a naive population, thus leading to the virus already spreading outside of the typical season.

Due to the very high infection rates 21/22 and high occupancy rates in pediatric infectious disease wards, there was much press discussion about a particularly severe RSV season, attributing this to a weakened immune system due to the lack of having to deal with infections while isolation measures were in place. Our data, however, reflect a catch-up effect with high patient numbers but no increase in disease intensity; comparable results are reported by Betts et al. (37). This is consistent with a normalization of epidemiology in recent years, with a later onset of the RSV season, higher disease severity, and decreasing incidence (31).

As criteria for the severity of infections, we refer to the higher proportion of oxygen required and the more frequent need for intensive care, as well as the occurrence of bacterial superinfections. For example, in season 2022/23 about 46% of our hospitalized children required oxygen therapy (Figure 3), a proportion which is in line with recent literature data (44). The need for intensive care was also highest in season 2022/23, a substantial increase, also reported by different other authors (36, 45, 46). Bacterial superinfections increased markedly in 2022/23 and after (Table 1), which can be attributed most likely to the relaxation of different hygiene measures at the end of the pandemic (47).

In contrast to a recent Polish study, where hospital stay in RSV patients decreased from 10 to 8 days (pre- compared to post-COVID-19) (48), our differences in overall length of stay could not be used as a criterion, as the very high number of infections in the catch-up season forced us to discharge some of the children from inpatient care very early. Moreover, short in-patient treatment is forced by the German DRG-system, i.e., reimbursement for hospitals is based on diagnostic related groups (DRG), not on the duration of hospital stay.

In this context, the question arises whether this lack of care capacity represents a risk to healthcare in the future during times of any kind of increased infection, and whether the maintenance of reserve wards and staff is a necessary consequence of this experience.

Lastly, it has been shown that unforeseeable factors can lead to significant deviations from normal infection seasons with strongly fluctuating patient numbers and seasonal occurrence, to which the healthcare system should be able to respond adequately.

Looking at the affected age groups, it was initially assumed that there were more older children among our patients in the catch-up season 2021/22 compared to the pre-COVID years. This appeared to be interesting with regard to the general assumption that the most severe RSV infections occur in patients below 2 years of age. But due to new hygiene recommendations and an increasing number of admissions we changed our testing strategy in 2021, i.e., testing was expanded to older age groups. Before, mainly children up to two years of age with appropriate symptoms were tested. Due to this bias, we only consider patients under 2 years of age in our analysis. However, our widespread testing led us to rethink our approach, as we were able to demonstrate that a significant proportion of hospital admissions in older children also occur due to RSV infection. This may also be of interest for future vaccination strategies and cohort studies.

After the start of the pandemic no more nosocomial RSV infections have occurred in our hospital. This could be accounted for the expanded testing and stricter isolation measures and cohorting, as well as for the broader awareness among the population regarding hygiene compliance measures, including visitors to the hospital. But restrictively it must be mentioned, that there was no follow up of discharged patients. Therefore, we can only refer to nosocomial infections that occurred while the patients were still hospitalized.

An interesting factor for the assessment of upcoming RSV seasons will be the numbers of immunization against the RS Virus after indication was expanded to all infants experiencing their first RSV season. Either maternal RSV vaccination during pregnancy or long-acting RSV monoclonal antibodies for infants are available (49). Intensive nirvesimab vaccination programs have already been started in Germany, whereas the acceptance of maternal vaccination still is an interesting question for the future.

In the past season 24/25, after the beginning of the immunization program in maternity clinics and in pediatricians’ practices particularly of newborns, the number of young infants among our inpatients had already begun to decrease. This could already be an initial result of this measure, although it is not reflected in our figures yet. But the first trend from Germany for 2024/25 indicates a decrease of roughly 50% in reported RSV cases in infants (31). Data from the US, where maternal RSV immunization and administration of nirsevimab to infants already started in 2023, suggest also a high effectivity of these prophylactic measures; two different surveillance networks indicate a decrease of RSV-associated hospitalization rates in infants aged 0–7 months of 28% and 43% compared with the rates of the pre-COVID seasons (50).

In conclusion, our study shows that the usual RSV epidemiology of inpatient care abruptly changed with the start of the COVID-19 epidemic. After a loss of severe RSV associated disease in 2020/21 a catch up effect could be notified in hospitalized patients in the following seasons, ending up in an almost normal season 2024/25. Future RSV monitoring is strongly recommended to calculate the hospital necessary resources, since vaccination programs, i.e., nirsevimab for infants and maternal vaccination during pregnancy, have already been started in Germany.

## Limitations

We performed a retrospective evaluation of hospital data charts without a pre-defined study protocol; thus, some limitations need to be mentioned.

Diagnosis and treatment of RSV infection followed our hospital SOPs; these were influenced by personal decisions of the physicians and they also slightly changed during the study period due to new guidelines. Nevertheless, we didn't notice significant changes in the outcome.

In general, the cut off for oxygen supplementation was an oxygen saturation lower than 90%. But individual case management sometimes required a modification of this threshold. For example, in patients with higher CO_2_-retention or severe airway obstruction oxygen supplementation started earlier, i.e., at saturation levels of 90%–92%, since oxygen also acts as an anti-obstructive medication. This potential bias is due to the retrospective study design, but it is minimized by the ongoing SOP and the consistency of our health personnel.

The indication for intensive care was the need for respiratory support due to an unstable respiratory situation with oxygen supplementation of more than 4–5 L/h and/or CO_2_ retention of more than 70%. This should be kept in mind when comparing our results to other studies based on clinical data with different indications for ICU admission.

All information of hospitalized patients with RSV infections included in the study was taken from the hospital charts and the discharge letter. There was no follow up of patients after discharge. Therefore, we can only refer to nosocomial infections that occurred while patients were hospitalized.

Concerning a suspected shift towards older children in the years during and after the pandemic, interpretation of our data is limited, since children aged 2 years and older were excluded from our analyses (see method section). Looking at our post-pandemic data when we tested older children too, we found a shift in age, from a median age of 7.6 months in the season 21/22 to 12.5 months in the season 2024/25. This shift of age is probably due to the start of the wide immunization of young infants and newborns in Germany. Further studies could address this finding when looking at immunization strategies for older children, especially with comorbidities.

## Data Availability

The data analyzed in this study is subject to the following licenses/restrictions: sensitive patient data. Requests to access these datasets should be directed to Wolfgang Kamin: wolfgang.kamin@hamm.johanniter-kliniken.de.
